# Inversion-recovery ultrashort-echo-time (IR-UTE) MRI-based detection of radiation dose heterogeneity in gynecologic cancer patients treated with HDR brachytherapy

**DOI:** 10.1186/s13014-024-02499-2

**Published:** 2024-08-06

**Authors:** Khadija Sheikh, Bruce L. Daniel, Michael Roumeliotis, Junghoon Lee, William T. Hrinivich, Thomas Benkert, Himanshu Bhat, Ravi T. Seethamraju, Akila N. Viswanathan, Ehud J. Schmidt

**Affiliations:** 1 Department of Radiation Oncology, Johns Hopkins University School of Medicine, 5255 Loughboro Road NW, Washington, DC USA; 2 Department of Cardiology, Johns Hopkins University School of Medicine, Baltimore, MD USA; 3 Department of Radiology, Stanford University, Stanford, CA USA; 4 MR Application Predevelopment, Siemens Healthineers AG, Erlangen, Germany; 5 Siemens Healthineers, Boston, MA USA

## Abstract

**Purpose:**

To evaluate the relationship between delivered radiation (RT) and post-RT inversion-recovery ultrashort-echo-time (IR-UTE) MRI signal-intensity (SI) in gynecologic cancer patients treated with high-dose-rate (HDR) brachytherapy (BT).

**Methods:**

Seven patients underwent whole-pelvis RT (WPRT) followed by BT to the high-risk clinical target volume (HR-CTV). MR images were acquired at three time-points; pre-RT, post-WPRT/pre-BT, and 3–6 months post-BT. Diffuse-fibrosis (F_Diffuse_) was imaged with a non-contrast dual-echo IR (inversion time [TI] = 60 ms) UTE research application, with image-subtraction of the later echo, only retaining the ultrashort-echo SI. Dense-fibrosis (F_Dense_) imaging utilized single-echo Late-Gadolinium-Enhanced IR-UTE, acquired ∼ 15 min post-Gadavist injection. Resulting F_Diffuse_ and F_Dense_ SI were normalized to the corresponding gluteal-muscle SI. Images were deformably registered between time-points based on normal tissue anatomy. The remnant tumor at both time-points was segmented using multi-parametric MRI. Contours corresponding to the 50%, 100%, 150%, and 200% isodose lines (IDLs) of the prescription BT-dose were created. Mean F_Diffuse_ and F_Dense_ SI within (i) each IDL contour and (ii) the remnant tumor were calculated. Post-BT F_Diffuse_ and F_Dense_ SI were correlated with prescribed BT-dose. To determine the relationship between BT-dose and IR-UTE SI, the differences in the post-BT F_Dense_ across IDLs was determined using paired t-tests with Bonferroni correction.

**Results:**

F_Dense_ was higher in regions of higher dose for 6/7 patients, with mean ± SD values of 357 ± 103% and 331 ± 97% (*p* = .03) in the 100% and 50% IDL, respectively. F_Dense_ was higher in regions of higher dose in the responsive regions with mean ± SD values of 380 ± 122% and 356 ± 135% (*p* = .03) in the 150% and 50% IDL, respectively. Within the segmented remnant tumor, an increase in prescribed dose correlated with an increase in F_Dense_ post-BT (*n* = 5, *r* = .89, *p* = .04). Post-BT F_Diffuse_ inversely correlated (*n* = 7, *r* = -.83, *p* = .02) with prescribed BT-dose within the 100% IDL.

**Conclusions:**

Results suggest that F_Dense_ SI 3–6 months post-BT is a sensitive measure of tissue response to heterogeneous BT radiation-dose. Future studies will validate whether F_Diffuse_ and F_Dense_ are accurate biomarkers of fibrotic radiation response.

**Supplementary Information:**

The online version contains supplementary material available at 10.1186/s13014-024-02499-2.

## Introduction

Fibrosis is known to clinically develop as a late sequelae of radiation therapy. Early detection of fibrosis during radiotherapy with non-invasive imaging may allow for radiation treatment plan adaptation. Currently, limited tools exist to longitudinally monitor varying grades of fibrosis that are present during radiotherapy.

Small animal imaging using atomic-force microscopy, performed after radiation-dose administration, has shown that increased dose results in faster deposition of fibrotic molecules, as well as in a change of the fibrotic structural arrangement (“packing”). After a critical level of accumulation, there is a remodeling of the Type III collagen into primarily densely-packed Type I collagen structures (1). We introduce the terms diffuse and dense fibrosis to differentiate between organized collagen Type III layers and densely-packed Type I collagen structures, respectively.

The relationship between fibrosis and dose has previously been studied [1, 2]. It is well known that the incidence and the severity of lung fibrosis vary with the total biological dose, which is a function of total dose and dose per fraction [3, 4]. Changes in lung tissue density were shown to be strongly correlated with physician-identified radiographic fibrosis. In turn, the lung tissue density correlated with increasing dose to the lung in a conventional external beam radiotherapy (EBRT) setting. Pelvis radiotherapy also results in tissue atrophy and fibrosis [5, 6], where increasing dose leads to increased fibrosis accumulation [7]. Specifically, studies have demonstrated that fibrosis accumulation is accelerated in the bladder [7, 8] and rectum at higher radiation doses [7].

It should also be noted that delineation of fibrosis on CT images is difficult. Fibrosis volumes usually do not have sharply defined edges; this is especially true for lower-grades of deposition. MR based identification of fibrosis may allow for identification of low grade fibrosis as it is increasingly being used to evaluate cystic fibrosis [9, 10] and idiopathic pulmonary fibrosis [11].

It has been shown that ordered collagen layers have an ultrashort T2* relaxation time (< 1 ms at 1.5T) [1] suggesting that lower grades of fibrosis (diffuse fibrosis, i.e. F_Diffuse_) may be imaged by ultrashort echo time (UTE) MRI. In fact, UTE MRI methods have been utilized to image idiopathic pulmonary fibrosis [12]. Higher grades of fibrosis (dense fibrosis, i.e. F_Dense_) and regions of necrosis have been previously imaged using late-gadolinium-enhanced (LGE) inversion recovery (IR) MRI, such as the imaging of heart necrosis [13, 14], which involves imaging typically performed greater than 10 min post contrast-injection, where the inversion-pulse and the long delay after injection increases the fibrosis contrast relative to the other tissue components. In mouse models that received radiation therapy, regions that demonstrated late contrast enhancement corresponded histologically to intra-tumoral necrosis [15]. LGE MRI has also been used to evaluate radiation induced fibrosis in the prostate [16].

However, limited work has utilized UTE and LGE MRI to evaluate varying grades of radiation induced fibrosis (i.e. F_Diffuse_ and F_Dense_) in gynecologic cancer patients; specifically those receiving a highly spatially heterogeneous dose in a short period of time with brachytherapy (BT).

Using MRI to understand the formation of fibrosis as it relates to dose deposition, may allow for improved monitoring of tumor response, normal tissue toxicities, and radiotherapy plan adaptation. Specifically, these tools may help localize regions of the tumor that may benefit from dose escalation. Therefore, in the current study, we studied the relationship between non-contrast and LGE IR-UTE signal intensity (SI) and high-dose-rate (HDR) BT dose distribution. We hypothesized that (i) regions receiving a higher dose would also exhibit higher LGE IR-UTE SI levels (i.e. F_Dense_) post-BT. However, (ii) lower LGE IR-UTE SI (F_Dense_) would be found in regions in which there was reduced response to the radiation dose, such as pockets of persisting live tumor post-radiation (i.e. remnant tumor).

## Methods and materials

### Patient selection and inclusion criteria

Ten patients were enrolled in the study, from 2020 to 2023, of which seven completed the entire course of the study. Post-BT images were not acquired for three patients and, as a result, are not reported herein. All participants provided written informed consent to a study protocol approved by a local Research Ethics Board. All participants with gynecologic cancer underwent whole pelvis radiation therapy (WPRT) and HDR BT. The inclusion criteria included: any patient eligible for BT internal implantation without MR guidance, life expectancy of greater than 6 months, and ECOG performance status of < 2.

### Radiation treatment

All patients received WPRT of 45 Gy in 25 fractions (i.e. 1.8 Gy/fraction) using VMAT on an Elekta VersaHD LINAC (Elekta AB, Stockholm, Sweden). The HDR brachytherapy procedure was performed with the intent to comply to the American Brachytherapy Society consensus guidelines, with the most common physical dose prescription being 27.5 Gy in 5 fractions [17, 18]. The Venezia applicator (Elekta AB, Stockholm, Sweden) or the Syed template were used for treatment. In this cohort, patients were treated in the inpatient setting with either one procedure or two separate procedures one week apart. The treatment characteristics for each patient, including CTV D90Gy and the number of insertions are reported in Table 1.

**Table 1:** Patient summary, including disease site, stage, number of insertions, BT prescription dose and number of fractions, and total EQD2 (WPRT + BT) CTV D90

Subject	Response	Site	Stage	# of Insertions	BT Rx	Total EQD2 (WPRT + BT) CTV D90 (cGy)
Patient 1*	Y	Cervical	IIB	2	2500 cGy in 4 fx	7200
Patient 2**	Y	Vaginal	IIB	1	2000 cGy in 4 fx	7220
Patient 3	N	Cervical	IIB	1	2750 cGy in 5 fx	7770
Patient 4	Y	Cervical	IIIC2	1	2400 cGy in 4 fx	8080
Patient 5	Y	Cervical	IIB	2	2600 cGy in 4 fx	8380
Patient 6	N	Cervical	IV	2	2750 cGy in 5 fx	7390
Patient 7	Y	Vaginal	II	1	2750 cGy in 5 fx	7980

*LGE IR-UTE imaging post-BT not acquired; **LGE IR-UTE imaging 6 months post-BT

### Image acquisition

MR images were acquired at three time points baseline/pre-RT, post-WPRT/pre-BT (one day after delivery of the prescribed WPRT-dose), and 3 or 6 months post-BT on 1.5T MRI scanners (either MAGNETOM Aera or MAGNETOM Sola, Siemens Healthineers GmbH, Erlangen, Germany) using torso and spine phased-array coils. No patient was scanned on multiple MRI systems.

IR-UTE MRI was performed using a stack-of-spirals dual-echo research application sequence [19]. Whole pelvis IR-UTE images were acquired in the coronal plane (parameters: echo times [TE_1_, TE_2_] /repetition time (TR)/flip-angle(Ɵ) = 0.05 ms, 2.69 ms/7 ms /8º, field-of-view [FOV] = 36–39 cm, time-duration/spiral = 1800µs, 220 spirals/image, signal-averages [AV] = 1, resolution = 0.9 × 0.9 × 2.5 mm^3^ or 1.0 × 1.0 × 2.5 mm³, 88 slices/scan with 50% sice under-sampling, scan-time [TA] = 246s).

Non-contrast IR-UTE images were acquired to evaluate F_Diffuse_ with T_Inversion_ = 60 ms, with the inversion pulse used to null the fat signal and accentuate short T1 relaxation-time tissue components, while the LGE-IR-UTE images were acquired to evaluate F_Dense_ with T_Inversion_ = 200 ms, primarily to suppress vascular signal, approximately 15 min following contrast administration. All participants received a 0.1 mmol/kg dose of Gadavist (Gadobutrol, Bayer Radiology) via a power-injector. Herein we refer to the non-contrast IR-UTE images as F_Diffuse_ images and the LGE-IR-UTE images as F_Dense_ images.

The residual pockets, herein referred to as the remnant tumor, were detected reliably with multi-parametric MRI (mpMRI) as previously shown [20, 21]. To detect the remnant living tumor(s) volume at the time-points before (pre-RT), after WPRT (pre-BT), and 3–6 months post-BT, 2D T2 weighted turbo spin-echo (TSE) images were acquired along axial and sagittal planes (parameters: TE/TR/Ɵ = 122 ms/3760 ms/120º, resolution = 1 × 1 × 2.5 mm^3^, bandwidth [BW] = 300 Hz/pixel, echo train length [ETL] = 14, AV = 2, TA = 379 s). Axial diffusion-weighted images (DWI) were acquired using a single-shot fat-suppressed SE-EPI sequence with two sagittal saturation slabs placed at the left and right 27 cm-FOV borders along the phase-encoding direction to prevent folding and reduce geometric distortion (parameters: monopolar diffusion-encoding, diffusion-directions = 3, b-values = 100, 500, 900 s/mm^2^, TE/TR/Ɵ = 92 ms/6000 ms/90^º^, ETL = 158, resolution 1.7 × 1.7 × 3.7 mm^3^, BW = 106 Hz/pixel, TA = 596s). Additionally, Dynamic Contrast Enhanced (DCE) fat-suppressed 3DGRE images were acquired continuously from 1 min before contrast injection to 5 min after contrast injection (parameters: TE/TR/Ɵ = 1.97 ms/4.5 ms/20º, resolution = 1.3 × 1.3 × 2.0mm^3^, BW = 400 Hz/pixel, accelerated quick fat-sat, centric-encode, average = 1, 60 slices/slab, TA = 20 s/dynamic-frame).

### Analysis

**Fig. 1:**
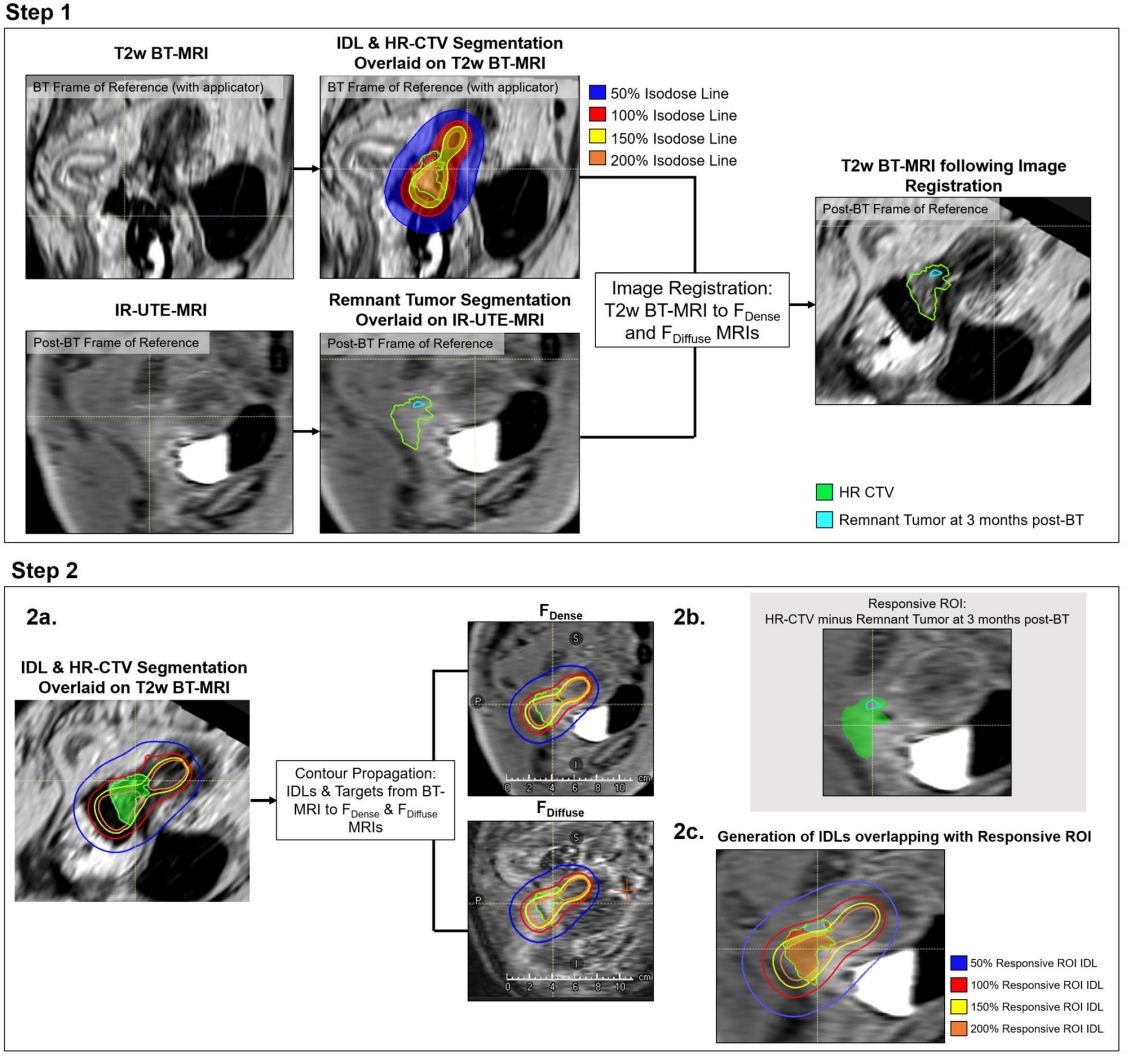
Flow charts outlining the study workflow. Step 1 demonstrates image registration and the contour propagation workflow. Step 2 shows the creation of the responsive ROI and isodose line (IDL) rings

Remnant tumor segmentation was performed on the mpMRI data images by a radiologist (BD), with over 25 years abdomino-pelvic cancer experience. The baseline/pre-RT tumor was contoured on the baseline/pre-RT MR images. The remnant tumor present post-WPRT/pre-BT was contoured on the post-WPRT/pre-BT and post-BT images. To reduce the uncertainty introduced from image registration, the remnant tumor present post-BT was contoured on the post-BT images only. The high risk clinical target volume (HR-CTV) was contoured by an experienced radiation oncologist. T2-weighted (T2w) images acquired at the time of BT (which were used for BT treatment planning) were registered to the F_Diffuse_ and F_Dense_ images acquired without the applicator post-BT (Fig. 1, Step 1). RayStation 2023B (RaySearch, Stockholm, Sweden) was used for biomechanical deformable image registration [22]. Briefly, the deformation is driven by controlling structures (i.e. uterus and cervix) that are defined in both the moving and the stationary image sets. Linear, elastic material properties are assigned based on structural information [23]. For patients receiving more than a single applicator insertion, each dose distribution was converted to equivalent dose in 2 Gy fractions (EQD2), and a composite dose volume was created in Raystation. As outlined in the ABS consensus guidelines on HDR BT for locally advanced cervical cancer [17], we utilized an alpha/beta ratio of 10 Gy for the target and 3 Gy for the OARs. An alpha/beta ratio of 10 Gy was used for fibrosis that formed in the target and an alpha/beta ratio of 3 Gy was used for fibrosis that formed elsewhere. The images acquired during the second insertion were deformably registered to the images acquired during the first insertion. This deformation field was applied to the dose and a sum dose volume was created.

Contours corresponding to the 50%, 100%, 150%, and 200% isodose lines (IDLs) of the prescription BT-dose were generated on the T2-weighted images acquired at the time of BT. IDLs were used and scaled relative to prescription to allow for consistency across patients that had varying BT prescriptions. As shown in Fig. 1, Step 2b, the responsive irradiated region, the region where remnant tumor was not detected post BT, was determined by subtracting the post-BT remnant tumor from the post-WPRT/pre-BT HR-CTV. IDL rings were generated to encompass a dose range as follows: 50% Ring = 50–100% of prescription; 100% Ring = 100–150% of prescription, 150% Ring = 150–200% of prescription, 200% Ring > 200% of prescription. We also generated dose rings to encompass the following four dose ranges: 10–25 Gy, 25–40 Gy, 40–55 Gy, and over 55 Gy. The “responsive ROI IDLs” were defined as the overlap between the responsive ROI and the IDL/dose rings. This was done for all four IDL rings (Fig. 1, Step 2c).

The non-contrast and LGE-IR-UTE images were normalized based on the mean SI of a 1 cm diameter region-of-interest (ROI) in the gluteal-muscle (i.e. normalized as a percent of gluteal muscle SI), which is outside the prescribed irradiated region as well as a tissue with uniform fibrosis. We define the mean F_Diffuse_ SI and F_Dense_ SI as the mean normalized SI derived from the non-contrast and LGE-IR-UTE images, respectively. Mean F_Diffuse_ and F_Dense_ SI were computed for the baseline/pre-RT, post-WPRT/pre-BT remnant tumor, post-BT remnant tumor, and responsive regions. The mean F_Dense_ SI within the IDL rings were also determined. We further computed the mean F_Dense_ SI within the “responsive ROI IDLs”. Table 2 provides a summary of ROIs utilized for analysis.

**Table 2:** Summary of MR images utilized for analysis and corresponding ROIs

Metric	ROIs	Purpose
Mean F_Diffuse_ SI from IR-UTE MR images	• IDL: 50%, 100%, 150%, 200% • Responsive ROI IDL: 50%, 100%, 150%, 200% • Pre-RT Tumor, Pre-BT Remnant Tumor, Post-BT Remnant Tumor • HR CTV	• IDLs: Determine the relationship between dose and F_Diffuse_ in the entire irradiated tissue • Responsive ROI IDLs: Determine the relationship between dose and F_Diffuse_ in the region of the tumor that responded to radiation • Tumors: Determine the relationship between dose and F_Diffuse_ in regions of the tumor that do not respond to radiation • HR-CTV: Determine the relationship between dose and F_Diffuse_ in the HDR BT irradiated target volume
Mean F_Dense_ SI from LGE-IR-UTE MRI	• IDL: 50%, 100%, 150%, 200% • Responsive ROI IDL: 50%, 100%, 150%, 200% • Pre-RT Tumor, Pre-BT Remnant Tumor, Post-BT Remnant Tumor • HR CTV	• IDLs: Determine the relationship between dose and F_Dense_ in the entire irradiated tissue • Responsive ROI IDLs: Determine the relationship between dose and F_Dense_ in the region of the tumor that responded to radiation • Tumors: Determine the relationship between dose and F_Dense_ in regions of the tumor that do not respond to radiation • HR-CTV: Determine the relationship between dose and F_Dense_ in the HDR BT irradiated target volume

### Statistics

Univariate relationships were evaluated using linear regressions (r^2^) and Pearson correlations (r), specifically to evaluate the relationship between dose delivered and the F_Dense_ SI of the irradiated remnant tumor and the F_Diffuse_ SI of the 100% Ring. Paired t-test with Bonferroni correction was performed to evaluate the difference between SI within the IDL contours. All statistical tests were performed using SPSS 28.0 (IBM, Armonk NY). Results were considered significant when the probability of making a Type I error was less than 5% (*P* < .05).

## Results

### Study subjects and dosimetry

Seven subjects who underwent WPRT (prescription of 45 Gy) followed by BT to the cervix or vagina were included. None of the seven patients had prior RT. Table 1 shows a patient summary, including disease site, staging, HDR applicator utilized, and the prescribed EQD2 BT dose.

### Pre- and post-BT SI within the remnant tumor and responsive regions

Quantitatively, the mean ± standard deviation pre-RT/pre-BT/post-BT F_Diffuse_ SI within the remnant tumor across all patients was 67 ± 31%/64 ± 28%/55 ± 22%, respectively. Across all patients with post-BT images, the mean ± standard deviation pre-RT/pre-BT/post-BT F_Dense_ SI *within the tumor* was 227 ± 59%/372 ± 155%/324 ± 76%, respectively; whereas the post-BT F_Dense_ SI *within the responsive* ROI was 355 ± 122%. Taken together, this suggests that the dense fibrosis is lower in regions of the remnant tumor compared to regions that receive the same dose and respond to radiation.

**Fig. 2:**
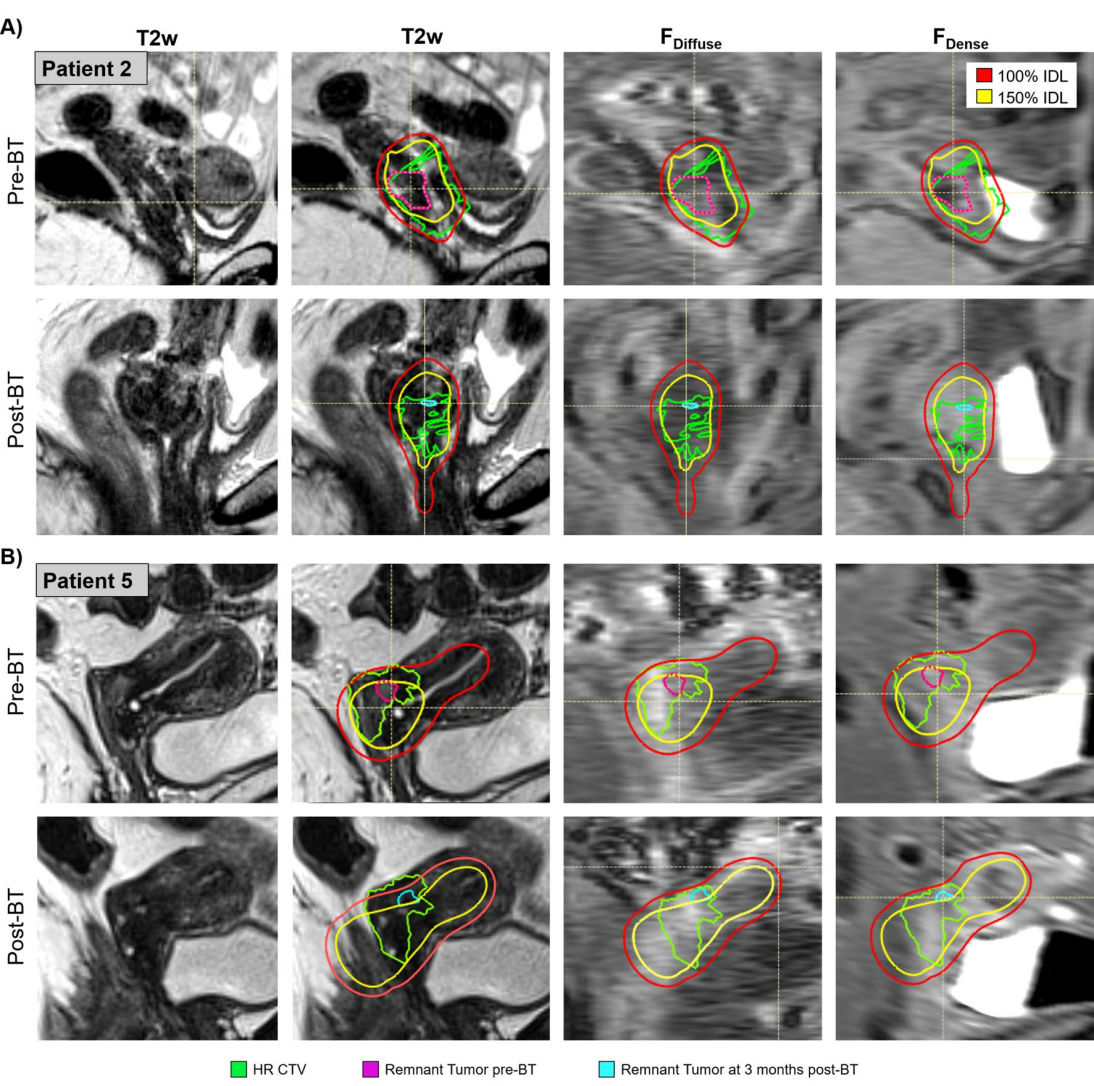
T2w, F_Diffuse_ and F_Dense_ images observed in two patients, Patient 2 (**A**) and Patient 5 (**B**), at two time points Pre-BT (top row) and post-BT (lower row) versus the radiation dose delivered. The F_Diffuse_ and F_Dense_ images’ grey scale is kept identical to demonstrate the changes that occur between Pre-BT and Post-BT. The images are overlaid with the 100 and 150% BT IDL contours (red and yellow), the BT HR-CTV (green), as well as the remnant tumor pre-BT (pink) and smaller 3–6 months post-BT boundaries (cyan). Note the higher F_Dense_ SI post-BT in the HR-CTV

Two representative cases, Patient 2 and Patient 5, are shown in Fig. 2A and B, respectively, to better illustrate this finding. The pre- and post-BT MR images are shown with the 100% and 150% IDLs (from the BT plan) overlaid along with the HR-CTV on all images. The remnant tumor pre-BT is shown in purple and remnant tumor post 3–6 months BT is shown in cyan. For both patients, F_Dense_ SI increases within the irradiated tissue (which includes both the MRI-detected gross tumor volume and cervix/uterine tissue that may include microscopic tumor).

### Post BT IR-UTE SI vs. BT dose within the remnant tumor and responsive regions

**Fig. 3:**
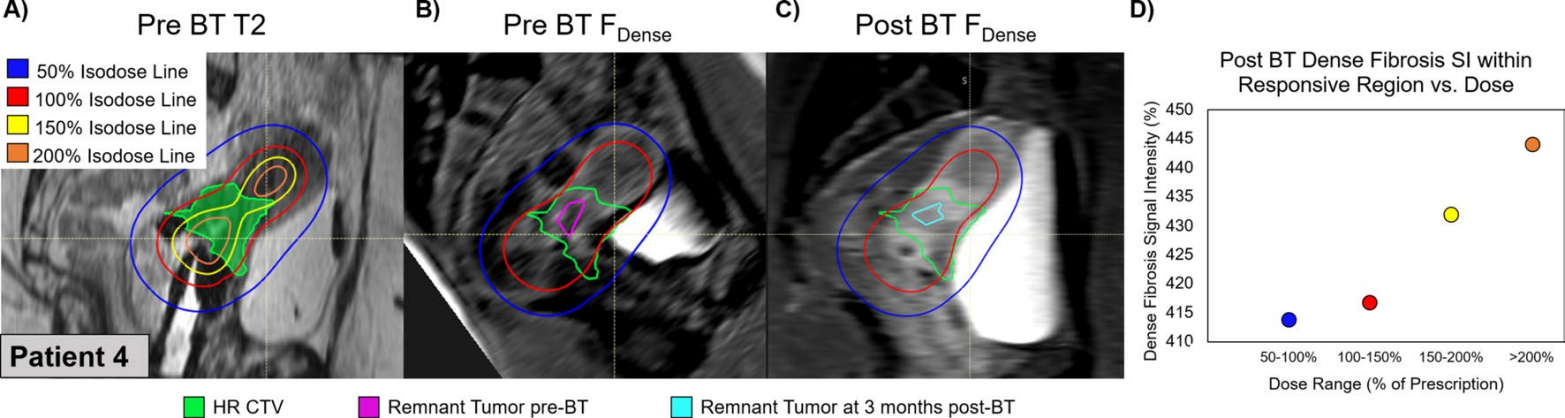
Fibrosis before and after BT versus BT dose for representative patient. (**A**) Anatomic T2-weighted images with color-overlaid BT-dose distribution shown for a representative patient. HR-CTV is shown in the green color wash. (**B**, **C**) F_Dense_ images pre-BT (**B**) and post-BT (**C**) shown with the 50%, 100% BT-isodose lines, overlaid in color with the prescribed HR-CTV (green), the pre-BT (purple) and the post-BT (blue) segmented remnant tumor. (**D**) Scatter plot showing the mean F_Dense_ SI within the “responsive region” HR-CTV, which excludes the remnant tumor post-BT, irradiated by the 50%, 100%, 150%, and 200% of BT-dose prescription

Figure 3 shows T2-weighted images (3A) acquired at the time of BT (with applicator) and F_Dense_ images pre- (3B) and post-BT (3C) in a single patient (Patient 4). The remnant tumor volumes pre-BT and 3-months post-BT are shown in purple and blue contours, respectively. It is visually apparent that the pre-BT F_Dense_ images have a lower SI than the post-BT F_Dense_ images in the region of the 100% IDL and within the HR-CTV (green). It is also interesting to note that the F_Dense_ SI within the *remnant tumor* at post-BT (cyan) appears to be of lower SI than the surrounding HR-CTV (green), which is the *responsive region*. As shown in the scatter plot (Fig. 3D), the F_Dense_ SI *within the responsive* ROI IDLs increases with dose.

**Fig. 4:**
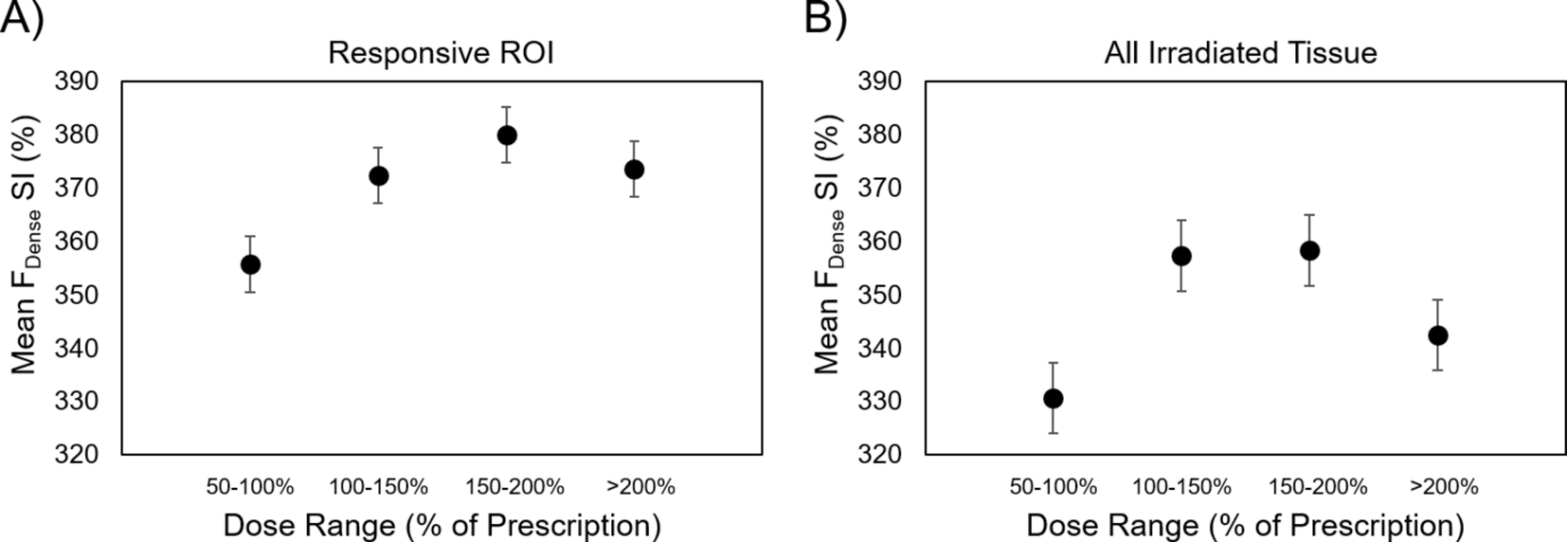
Quantitative dense fibrosis (F_Dense_) measured at 3–6 months post-BT versus administered BT dose in 6 patients. (F_Dense_) measured at 3–6 months post-BT versus administered BT dose in 6 patients. Mean F_Dense_, with error bars indicating the standard error, across all patients vs. isodose lines (50%, 100%, 150%, and 200% of prescription BT dose) within the (**A**) responsive ROI and (**B**) the irradiated tissue

Figure 4A shows the mean F_Dense_ SI *within the responsive* ROI IDLs as a function of dose for all patients. In six patients, as the delivered dose increased, the F_Dense_ SI increased. Specifically, the F_Dense_ SI peaked within the 150–200% dose range and then slightly decreased within the regions receiving greater than 200% of prescription dose. In responsive regions, dose was linearly correlated with dense fibrosis SI in regions receiving 100–150% of prescription dose (post-BT), although the relationship becomes non-linear and saturates at higher doses.

For all patients, Fig. 4B shows the post-BT F_Dense_ SI of the irradiated tissue (the HR-CTV and all normal tissue receiving dose), which includes both tumor and cervix/uterine tissue that may include microscopic tumor, within the IDLs as a function of dose. It can be seen that the SI within the 50% IDL (mean ± SD = 331 ± 96%) ring is significantly lower than the SI within the 100% IDL ring (mean ± SD = 357 ± 102%) (*p* = .02) and 150% IDL (mean ± SD = 358 ± 97%) (*p* = .05). Similar to the responsive ROIs, the irradiated tissue F_Dense_ increased with dose, but saturated around the 150–200% dose range. We should note that percent of prescription dose may be difficult to interpret with a mix of patients with different fractionations. Therefore, we also evaluated the F_Dense_ SI within absolute dose rings with ranges: 10–25 Gy, 25–40 Gy, 40–55 Gy, and > 55 Gy (Supplemental Fig. 1). Here, we noted that the SI within the 10–25 Gy ring (mean ± SD = 327 ± 90%) was significantly lower than the SI within the 40–50 Gy ring (mean ± SD = 358 ± 88%) (*p* = .05). We also noted that the F_Dense_ increased with dose for both the responsive ROI and all irradiated tissue, but saturated around 40 Gy for the responsive ROI. Interestingly, the F_Dense_ SI within the responsive region appears to be generally higher than the BT irradiated tissue. This may reflect increased fibrosis in tumor regions that were eradicated prior to the BT. In addition, we note that regions that received 200% of the BT prescription dose were found to not contain regions of remnant tumor post-BT.

Scatter plots for individual patients are shown in Supplemental Fig. 2. These plots show F_Dense_ of the responsive ROI IDLs measured at 3–6 months post-BT versus administered BT dose (as a percent of prescription). In general, the F_Dense_ within the responsive ROI IDLs increases with dose. However, this trend was not apparent for Patient 3 who had regions of high F_Dense_ SI pre-BT and in fact, had persistent disease following radiation treatment. Patient 2 appears to have F_Dense_ that saturated at higher doses. It should be noted that F_Dense_ images were only acquired for this patient 6 months (instead of 3 months) following BT and thus the SI may reflect later stages of recovery following RT.

**Fig. 5:**
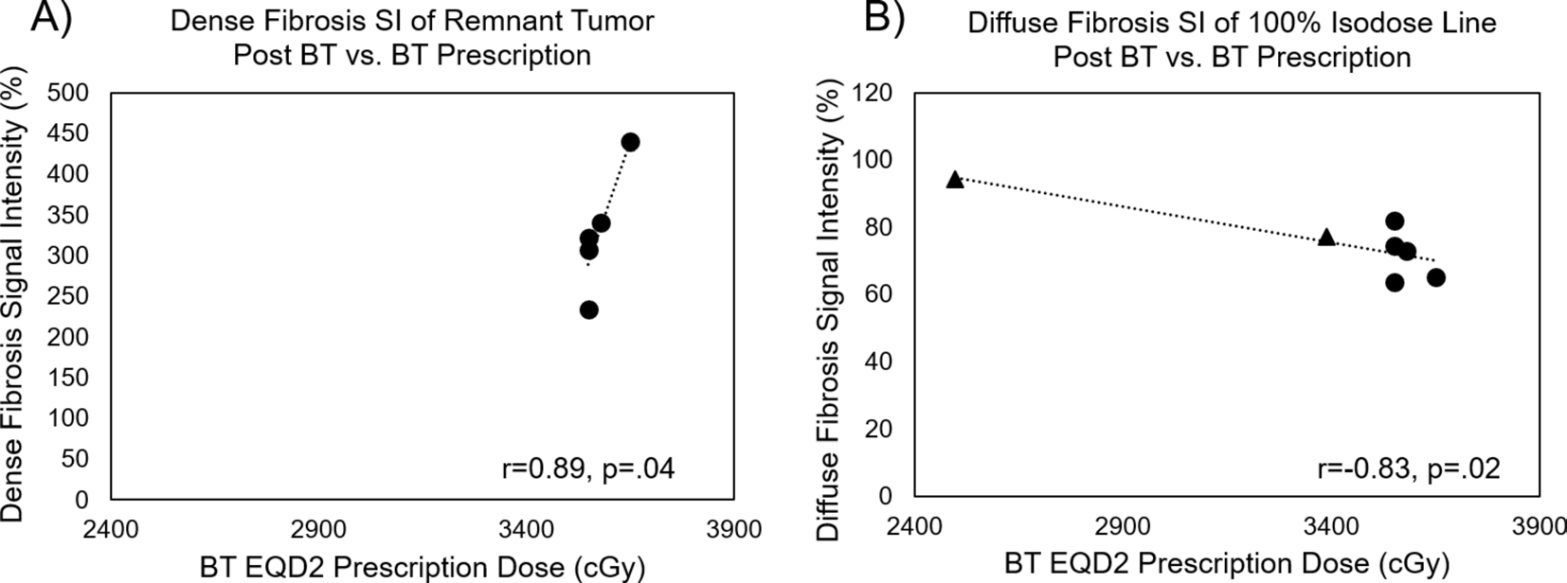
Correlations between the prescribed BT-dose and post-BT fibrosis. BT Dose vs. (**A**) post-BT dense-fibrosis SI within the remnant tumor, and (**B**) post-BT diffuse-fibrosis within the 100% IDL, which includes all irradiated tissue. Note that the diffuse-fibrosis signal intensities are lower than the dense-fibrosis intensities, suggesting fibrosis conversion to dense-fibrosis at higher dose levels, leaving diffuse fibrosis mostly at lower dose levels. Triangle markers indicate the two patients that did not have F_Dense_ images acquired 3 months post-BT

Scatter plots correlating dose with post-BT F_Diffuse_ and F_Dense_ SI are shown in Fig. 5 for 7 patients. Figure 5A shows that an increase in prescribed dose correlated with an increase in remnant tumor dense-fibrosis post-BT SI (*n* = 5, r = + 0.89, *p* = .04), indicating that F_Dense_ SI measures response to BT-dose. Figure 5B shows that a decrease in prescribed BT-dose correlated with an increase in the post-BT F_Diffuse_ SI within the 100% IDL (*n* = 7, *r* = -.83, *p* = .02), indicating that F_Diffuse_ post-BT is restricted to regions receiving relatively lower-dose. Overall, post-BT F_Diffuse_ SI was low relative to the F_Dense_ SI. This differs from data we collected in the course of WPRT, where the F_Diffuse_ SI was higher. At 3–6 months post-BT, the F_Diffuse_ SI is expected to be lower than F_Dense_, whereas at a short delay time post-RT (estimated at < 1month), the F_Diffuse_ is expected to dominate [24]. This suggests that by 3–6 months post-BT, there occurred a conversion of most of the diffuse fibrosis into dense fibrosis.

## Discussion

We utilized IR-UTE MRI to evaluate radiation response in gynecologic cancer patients undergoing HDR-BT. The main findings of this study are: (1) F_Dense_ increased post-BT, (2) at 3–6 months post-BT, F_Diffuse_ SI was lower than F_Dense_ SI and inversely correlated with dose, and (3) at 3–6 months post-BT, F_Dense_ SI correlated with BT dose in regions that responded to radiation.

This work was focused on determining the relationship between fibrosis creation and dose delivery during HDR BT where the dose delivery was (i) highly spatially heterogeneous and (ii) delivered over a short course of time. The relationship between EBRT fractionated dose delivery and fibrosis, which is more complex and thus may require frequent MRI imaging during RT, due to competing physiological processes, such as fibrosis creation versus clearance, will be the subject of a future manuscript. We should also note the characteristics of the EBRT sets the initial conditions for the BT procedure (i.e. the volume which needs to be irradiated and the regions within it that require more dose). In this study, this was considered through the HR-CTV, which relied on T2-weighted MRI, and the clinician prescribed radiated hot-spots. Processing of more MRI tissue biomarkers may change the prescribed BT dose distribution and may slightly change our results.

First, we observed that F_Dense_ increased following BT. This is consistent with the notion that large doses delivered during BT will lead to increased cell death which will ultimately be observed as an increase in fibrosis. Radiation injury also triggers inflammation and ultimately stimulates trans-differentiation of fibroblasts into myofibroblasts [5]. These myofibroblasts produce excess collagen and other extracellular matrix components. Consequently, these increases in collagen are reflected in an increase in MR derived F_Dense_ SI. In a porcine model where proton therapy was used to ablate the heart, gross fibrosis was observed using LGE-MRI 12 weeks following RT (40 Gy) [25].

We also showed that F_Diffuse_ was inversely correlated to dose at the post-BT time point. At this late time delay following BT, F_Diffuse_ is mostly present in regions which received a lower dose, while at higher dose regions, F_Diffuse_ has mostly converted into F_Dense_. This may reflect the changes in collagen structure due to its remodeling, since higher radiation dose leads to a higher concentration of collagen, which compacts [7, 26].

Finally, we observed that F_Dense_ correlated with dose within the entire irradiated regions, as well as within the remnant tumor region alone. This finding is similar to previous findings in the lung, where CT fibrosis delineation correlated with dose in small cell lung cancer patients receiving external beam RT [1]. This work [1] evaluated physician identified fibrosis and demonstrated that the intensity of fibrosis increased with increasing dose, when dose exceeded the threshold of 30–35 Gy. This is also similar to what was reported in the heart of irradiated porcine models [27] where the extent and intensity of fibrosis correlated with increasing dose, when it was above the threshold of 32.5 Gy. We note that the patients in our study received a cumulative dose of greater than 70 Gy (WPRT + BT).

Although this study provides promising preliminary results, we must point out the following study limitations. This study included a small cohort of patients and caution must be taken when extrapolating these results to a larger sample size. These results should be validated in a larger cohort. We should also note that target segmentation was performed by a single expert radiologist. The reproducibility of these methods will be evaluated with multiple observers in a larger cohort in a multi-center trial. In this study, we employ contrast-enhanced LGE IR-UTE imaging to detect F_Dense_. There may be difficulties in use of LGE in patients with poor urinary clearance rates. These patients may require that the LGE imaging be performed at later times post-injection. Another alternative to contrast injection is to perform non-contrast T1 mapping to detect dense fibrosis (which has a slightly longer T1 than pelvic soft tissues), although native T1 mapping requires longer scan times and has lower sensitivity to fibrosis. Finally, image registrations performed between images with the BT applicator to images without the applicator may result in inaccuracies due to the different properties of the applicator and tissue [28, 29].

Only two specific time points were imaged, with the 3–6 month time point expected to better reflect the maximal extent of F_Dense_. The sensitive relationship we observed between F_Dense_ and delivered dose may therefore reflect the culmination of most fibrosis deposition (caused by extracellular matrix fibrosis compaction and by cell death) by this time. Imaging performed early (days) after RT is expected to show a larger fraction of F_Diffuse_ and less F_Dense_ [24]. The time course of the development of F_Diffuse_ and F_Dense_ needs to be explored in more detail, in animal models, or by more frequent human imaging.

We observed a slight decrease in F_Dense_ in regions receiving more than 200% of prescription dose. This may be due to reduced contrast agent uptake in regions of low perfusion and very high necrotic cells. This requires further investigation.

Taken together, post-radiation fibrosis varies between the early developments of diffuse fibrosis, resulting from tumor shrinkage, wound healing, and the later development of dense scar, which results from fibrosis compaction and increasing cell death. This dense scar tissue may result in degradation of tissue function due to loss of tissue elasticity. The current study demonstrated the relationship between dose delivered and IR-UTE MR-based post-radiative fibrosis SI. The IR-UTE MRI tools identify fibrotic regions and possibly gauge tissue response to heterogeneous radiation distributions, whether delivered via brachytherapy or via external beam RT. Integrating UTE MRI into clinical practice may allow for improved therapeutic outcomes, reduced adverse effects, and tailored radiotherapy strategies.

### Electronic supplementary material

Below is the link to the electronic supplementary material.

Supplementary Material 1: **Fig. 1.** Quantitative dense fibrosis (F_Dense_) measured at 3–6 months post-BT versus administered BT dose in 6 patients. (F_Dense_) measured at 3–6 months post-BT versus administered BT dose in 6 patients. Mean F_Dense_, with error bars indicating the standard error, across all patients vs. dose range (10–25 Gy, 25–40 Gy, 40–55 Gy, and over 55 Gy of prescription BT dose) within the (A) responsive ROI and (B) the irradiated tissue

Supplementary Material 2: **Fig. 2.** Normalized LGE-IR-UTE MR images used for dense fibrosis quantification (F_Dense_) measured at 3–6 months post-BT versus administered BT dose in 6 patients. **In patient 2: the 6 month post-BT time point is shown. In *Patient 3 large amounts of fibrosis pre-BT were observed, so the fibrosis concentration may be saturated and therefore not respond to BT (which is also evident from the abnormally high F_Dense_ SI)

## Data Availability

The datasets used and analyzed during the current study are available from the corresponding author on reasonable request.
