# Supplementary figures and images for: Inversion-recovery ultrashort-echo-time (IR-UTE) MRI-based detection of radiation dose heterogeneity in gynecologic cancer patients treated with HDR brachytherapy
Source: Radiat Oncol. 2024 Aug 6;19:105. doi: 10.1186/s13014-024-02499-2 (PMC11305063; doi:10.1186/s13014-024-02499-2)

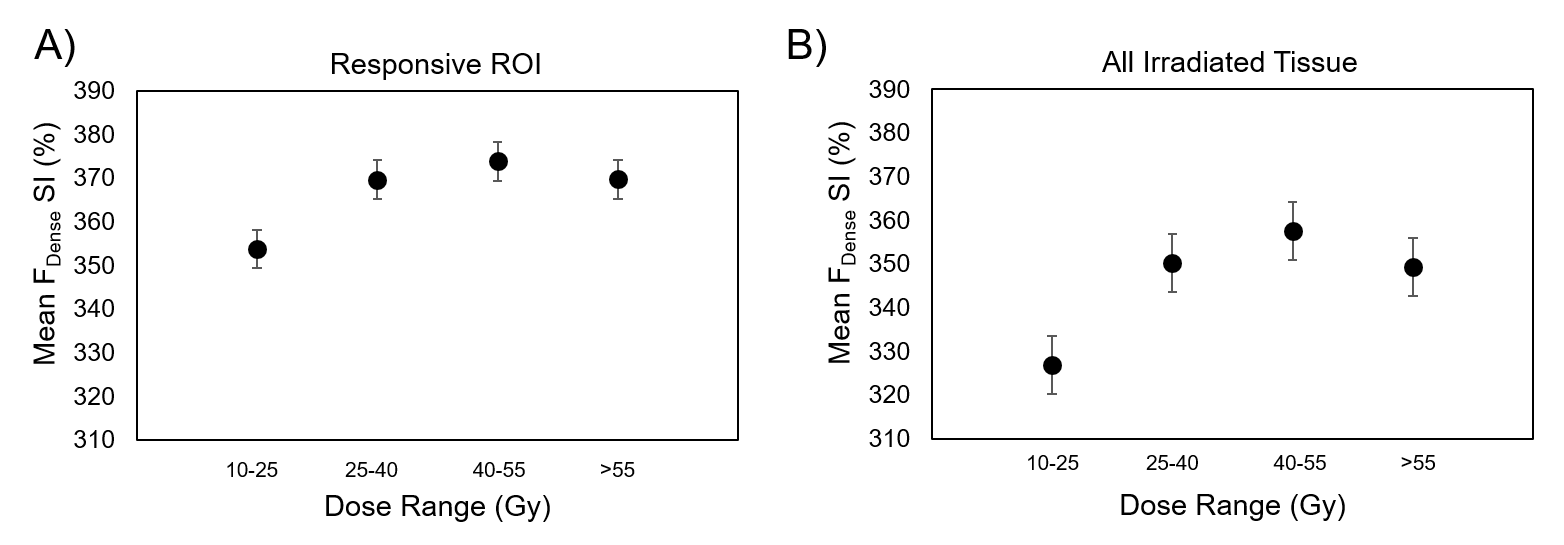

Supplement: Supplementary file 1 — Supplementary Material 1: Fig. 1. Quantitative dense fibrosis (FDense) measured at 3–6 months post-BT versus administered BT dose in 6 patients. (FDense) measured at 3–6 months post-BT versus administered BT dose in 6 patients. Mean FDense, with error bars indicating the standard error, across all patients vs. dose range (10–25 Gy, 25–40 Gy, 40–55 Gy, and over 55 Gy of prescription BT dose) within the (A) responsive ROI and (B) the irradiated tissue [file 13014_2024_2499_MOESM1_ESM.tif]

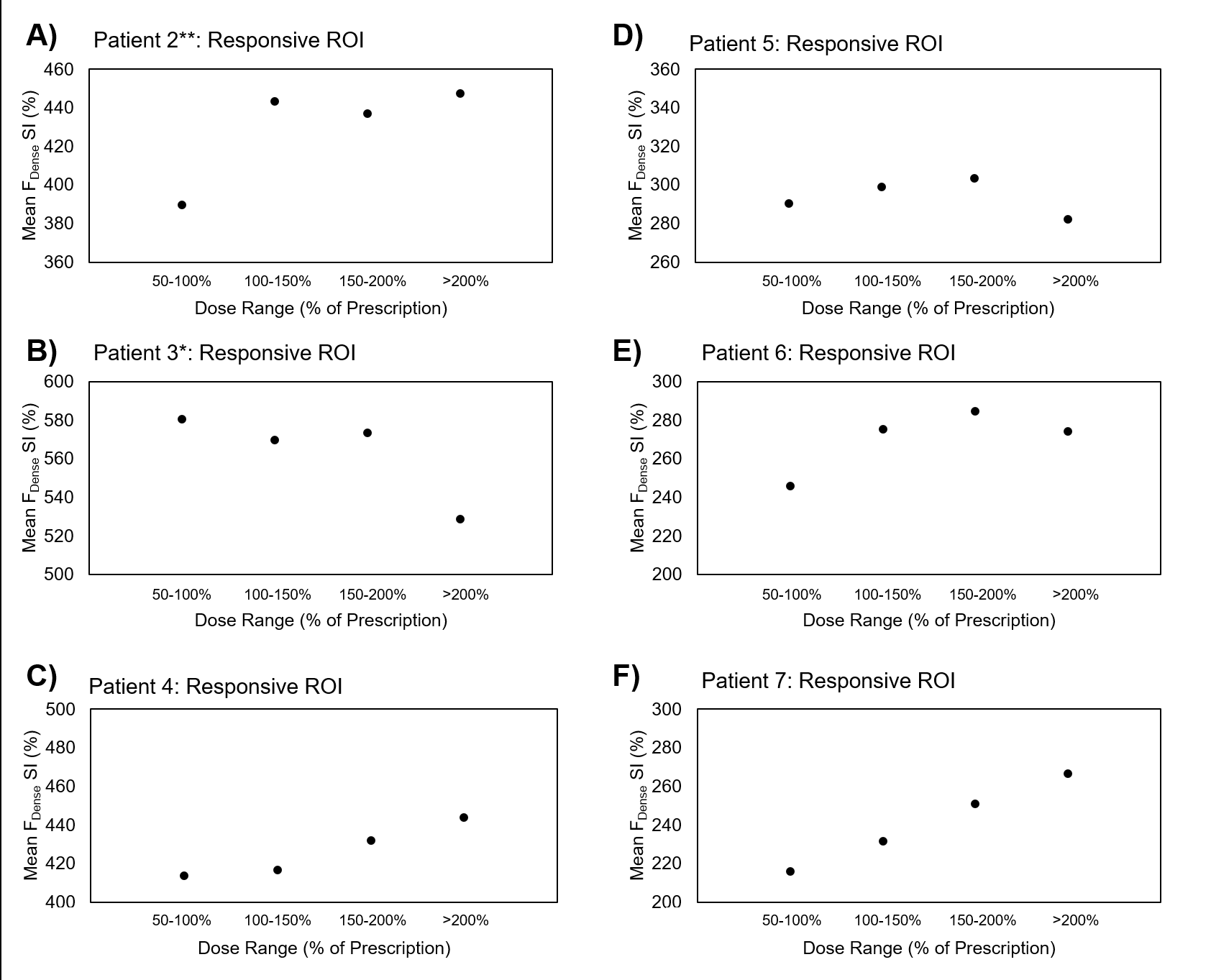

Supplement: Supplementary file 2 — Supplementary Material 2: Fig. 2. Normalized LGE-IR-UTE MR images used for dense fibrosis quantification (FDense) measured at 3–6 months post-BT versus administered BT dose in 6 patients. **In patient 2: the 6 month post-BT time point is shown. In *Patient 3 large amounts of fibrosis pre-BT were observed, so the fibrosis concentration may be saturated and therefore not respond to BT (which is also evident from the abnormally high FDense SI) [file 13014_2024_2499_MOESM2_ESM.tif]
